# Pathogenic analysis of sputum from ventilator-associated pneumonia in a pediatric intensive care unit

**DOI:** 10.3892/etm.2012.757

**Published:** 2012-10-22

**Authors:** BO-TAO NING, CHEN-MEI ZHANG, TAO LIU, SHENG YE, ZI-HAO YANG, ZHEN-JIE CHEN

**Affiliations:** Department of Pediatric Intensive Care Unit, Children’s Hospital, School of Medicine, Zhejiang University, Hangzhou, Zhejiang 310003, P.R. China

**Keywords:** pediatric intensive care unit, ventilator associated pneumonia, sputum, drug susceptibility

## Abstract

Ventilator-associated pneumonia (VAP) is a common and sometimes fatal complication in pediatric intensive care units (PICU). The aim of our study was to characterize the distribution and drug susceptibility of the pathogens isolated from the sputum of patients with VAP in the PICU of our hospital and to provide support to the administration of antibiotics early and reasonably in the clinic. Our study was conducted between January 2007 and December 2011 at the PICU of the Children’s Hospital of Zhejiang University School of Medicine. The endotracheal aspirates were collected and transported to a microbiology laboratory within 15 min. The pathogens were routinely analyzed and identified with Vitek 60 and Kirby-Bauer disk diffusion methods. Among the 121 VAP patients, 127 pathogenic strains were isolated from sputum specimens. Gram-negative and gram-positive bacteria and fungi accounted for 64.57% (82/127), 29.92% (38/127) and 5.51% (7/127), respectively. *Acinetobacter baumannii* (25.61%), *Escherichia coli* (20.27%), *Stenotrophomonas maltophilia* (20.27%), *Klebsiella pneumoniae* (16.22%) and *Pseudomonas aeruginosa* (9.46%) were frequently identified isolates among gram-negative bacteria. Staphylococci were susceptible to vancomycin and linezolid. All fungi were sensitive to the antimicrobial agents. The gram-negative bacteria were more prevalent than gram-positive bacteria and fungi in VAP and demonstrated a higher drug resistance. It is important to administer antimicrobial agents early and reasonably for children with VAP. Knowledge of antibiotic resistance and the characteristics of drug resistance is important for VAP prophylaxis and treatment.

## Introduction

Children in the pediatric intensive care unit (PICU) are at high risk of hospital-acquired infection. Ventilator-associated pneumonia (VAP) is the most common hospital-acquired infection, with an incidence of 8–28% (1,2). VAP is becoming a common and fatal complication as mechanical ventilation is being increasingly used in the PICU. VAP is defined as pneumonia occurring >48 h after patients have accepted intubation and received mechanical ventilation. It often presents as deteriorated pneumonia or as new foci in the lungs. The occurrence rate in adults reaches 9–27% and the mortality rate is 20–50% (3–5). In children, due to their poor immunity, serious original disease and prolonged mechanical ventilation, the mortality rate of 24–76% is higher than in adults (1,2). In order to study the epidemiology and changes in antibacterial susceptibility to allow guidance of the empirical and reasonable use of antibiotics to reduce the mortality of children with VAP, we retrospectively studied the pathogenic bacteria distribution and drug resistance in 121 VAP patients in the PICU of the Children’s Hospital affiliated to Zhejiang University from January 2007 to December 2011.

## Patients and methods

### Patients

From January 2007 to December 2011, a total of 492 patients underwent mechanical ventilation and of these, 121 patients developed VAP. From these patients 127 strains were isolated. The general data of the 121 children (whose ages ranged from 2 months to 16 years, 3 months; median age 3 years, 6 months) are shown in Table I. The present study was approved by the ethics committee of the Children’s Hospital of Zhejiang University School of Medicine and informed consent was obtained from all patients.

### Definition of VAP

The diagnostic criteria of VAP are as follows: i) pneumonia occurring more than 48 h after intubation and mechanical ventilation; ii) two or more of the following four criteria: a) fever >38.3°C; b) leukocytosis >10,000 cells/ml or <5,000 cells/ml; c) purulent tracheobronchial secretion; and d) new pathogenic bacteria isolated from bronchial secretions; iii) a new and persistent (>48 h) infiltrate on chest radiograph (6).

### Antibiotic susceptibility test

Bacteria were identified using a Vitek-60 automated analysis system which was purchased from bioMérieux (Durham, UK). Susceptibility paper and susceptibility medium (MH medium) were purchased from Oxiod Ltd. (Basingstoke, UK) and Marcel Mérieux, respectively. Under sterile conditions, a disposable sterile sputum collector was used to collect secretion samples from the trachea through an endotracheal intubation catheter. A total of 127 sputum samples were cultured and a routine drug susceptibility test was performed within 15 min using the Vitek-60 analysis system and Kirby-Bauer disk diffusion method. The extended-spectrum β-lactamase (ESBL) strains were detected by double disk test. If more than two consecutive results were the same in one patient, we recorded the results of the sputum culture once; if the results were different, we recorded each result of the sputum culture.

### Statistical analysis

All data were analyzed using Stata software, version 9.0. Measurement data are denoted by median values and numeration data were presented as rate and constituent ratio.

## Results

### Distribution of pathogens

The incidence of VAP was 24.59% (121/492). A total of 127 strains were detected from 121 VAP patients. Six patients were diagnosed with mixed infections. None were recurrent. Gram-negative bacteria, gram-positive bacteria and fungi accounted for 64.57% (82/127), 29.92% (38/127) and 5.51% (7/127) of the strains, respectively. The most common pathogens were *Acinetobacter baumannii, Escherichia coli, Stenotrophomonas maltophilia, Klebsiella pneumoniae and Pseudomonas aeruginosa* (Table II).

### Drug resistance results of common pathogens

The detection rates of ESBL-producing *Escherichia coli*, ESBL-expressing *Klebsiella pneumoniae* and methicillin-resistant *Staphylococcus aureus* (MRSA) were 58.82% (10/17), 41.67% (5/12) and 66.67% (4/6), respectively.

The gram-negative bacilli demonstrated multiple drug resistance. The 5 most commonly isolated strains were *Acinetobacter baumannii, Escherichia coli, Stenotrophomonas maltophilia, Klebsiella pneumoniae* and *Pseudomonas aeruginosa*. All of them, with the exception of *Acinetobacter baumannii*, were sensitive to cefoperazone/sulbactam*. Escherichia coli* and *Klebsiella pneumoniae* demonstrated no drug resistance to meropenem and imipenem; however, they had a high resistance to most penicillins and cephalosporins. All *Pseudomonas aeruginosa* isolates were sensitive to cefoperazone/sulbactam, imipenem, piperacillin/tazobactam, amikacin and levofloxacin; however, the drug resistance to meropenem was high (72.73%). All *Stenotrophomonas maltophilia* isolates were sensitive to cefoperazone/sulbactam, piperacillin/tazobactam and levofloxacin; 35.29% of them were resistant to cefoxitin and its drug resistance rate to other antibiotics was >70% (Table III).

Gram-positive cocci had no drug resistance to vancomycin and linezolid; however, they demonstrated high drug resistance to penicillin-G, erythromycin, clindamycin and sulfamethoxazole. The positive percentage of β-lactamase was 76.32% (29/38) in 38 staphylococci. The 10 *Staphylococcus epidermidis* strains were resistant to oxacillin. The resistance to oxacillin was 88.89% (8/9) in other coagulase negative staphylococcus (CoNS) strains; however, all 6 *Staphylococcus aureus* strains demonstrated no drug resistance to oxacillin (Table IV). The 7 strains of *Candida albicans* were all susceptible to fluconazole.

## Discussion

The PICU is the centre of intensive care and treatment for critically ill children and invasive and complex techniques and equipment, including tracheal intubation and mechanical ventilation, are used frequently. Hamid *et al*(2) reported that VAP occurrence was closely associated with the following factors: i) age of the patient <1 year; ii) emergent intubation and iii) persistent and long-time sedation. Patients in the PICU often suffer from severe pathophysiological disorders and hypo-immunologic function. Intubation and mechanical ventilation are essential to maintain normal ventilation and gas exchange. The ventilator guarantees normal oxygenation; however, the airway mucosal destruction and barotrauma increases the risk of pathogenic colonization and infection in the respiratory system. We separated 127 strains of pathogens from 121 sputum samples collected from the lower respiratory tract. Gram-negative bacteria were the dominant pathogens and among them, the most frequent pathogens were *Acinetobacter baumannii, Escherichia coli, Stenotrophomonas maltophilia, Klebsiella pneumoniae* and *Pseudomonas aeruginosa. Staphylococcus epidermidis* was the most common gram-positive pathogen.

In recent years, the non-fermenters such as *Acinetobacter baumannii* and *Stenotrophomonas maltophilia*, have become increasingly prevalent. They have been the main pathogenic bacteria in hospital-acquired infection, particularly in VAP (7,8). We report that non-fermenters accounted for 46.34% of the cases of VAP and among them the percentage of *Acinetobacter baumannii* was 25.61%. High drug resistance often occurred with *Acinetobacter baumanii* due to the high expression of AmpC enzyme, synthesis of OXA-23 carbapenemases, downregulation of the sensitivity of the efflux pump and production of penicillin-binding protein (PBP). At the same time, certain drug-resistant bacteria may transfer drug-resistant plasmids between each other, resulting in multi-drug resistance (9,10). The current study demonstrated that *Acinetobacter baumannii* is only partly sensitive to aminoglycosides, quinolones, cefotaxime, piperacillin/tazobactam and cefoperazone/sulbactam and 100% resistant to carbapenems. Therefore, imipenem and meropenem are not the preferred antibiotics for *Acinetobacter baumannii*. The drug resistance rates to imipenem and meropenem were higher in other non-fermenters. The resistance rates of *Pseudomonas aeruginosa* to imipenem and meropenem were 0 and 72.73%, respectively, and in *Stenotrophomonas maltophilia,* they were 70.59 and 100%, respectively. These results indicate that there may be a natural drug resistance to these two antibiotics (11,12). Therefore, it is necessary to stringently manage and control the use of carbapenems due to the decreased sensitivity of *Acinetobacter baumannii*, increased resistance of *Pseudomonas aeruginosa* and the natural drug resistance of *Stenotrophomonas maltophilia.*

The wide usage of β-lactam antibiotics in the clinic induces the production of ESBL in *Escherichia coli* and *Klebsiella pneumoniae*. ESBL may hydrolyze cephalosporin and transfect between bacteria, which promotes the transmission of drug resistance between pathogens (13). The present study revealed that the percentages of ESBL in *Escherichia coli* and *Klebsiella pneumoniae* were 58.82 and 41.67%, respectively. Although they were sensitive to carbapenems, aminoglycosides, quinolones and antibiotics combined with an enzyme inhibitor, they were highly resistant to penicillin and cephalosporin. The gram-positive coccus gained high resistance to penicillin; however, they were completely sensitive to vancomycin and linezolid. Therefore, vancomycin and linezolid are preferred in severe infection caused by *Staphylococcus aureus* and β-lactam, sulfamethoxazole, erythromycin and clindamycin antibiotics are no longer recommended in the treatment of severe gram-positive cocci infection (14,15).

Fungal infection accounted for 5.51% of the cases of VAP in the current study. *Candida albicans* was the prime pathogen identified in fungal VAP and was sensitive to the majority of antifungal drugs. The factors that may be associated with fungal infections in children are low immune function, long-term usage of broad-spectrum antibiotics, unreasonable usage of glucocorticoids and invasive surgery (16,17).

VAP acutely threatens patient survival in the PICU. Its pathogenic distribution and drug susceptibility are diverse across different centers; therefore, once VAP is diagnosed, the first choice is empirical antibiotic treatment according to previous data. As studies of drug susceptibility are reported, the choice of antibiotic may be performed more selectively.

## Figures and Tables

**Table I:** General data of the 121 VAP patients.

Variables	Number of patients	Rate (%)
Gender		
Male	75	61.98
Female	46	38.02
VAP occurrence		
<5 days	36	29.75
≥5 days	85	70.25
Basic diseases		
Severe pneumonia	39	32.23
Sepsis with ARDS	17	14.05
Central nervous system infection	29	23.97
Trauma	11	9.09
Drowning	9	7.44
Drug poisoning	7	5.79
Epilepsy	3	2.48
Asthma	2	1.65
Non-traumatic		
intracranial hemorrhage	4	3.31

VAP, ventilator-associated pneumonia; ARDS, acute respiratory distress syndrome.

**Table II:** Distribution of detected pathogens in the sputum of children with VAP.

Pathogen	Number of strains	Proportion (%)
Gram-negative bacteria	82	64.57
*Acinetobacter baumannii*	21	25.61
*Escherichia coli*	17	20.27
*Stenotrophomonas maltophilia*	17	20.27
*Klebsiella pneumoniae*	12	16.22
*Pseudomonas aeruginosa*	11	9.46
*Sphingomonas paucimobilis*	2	2.70
*Aeromonas hydrophila*	1	1.35
*Salmonella thompson*	1	1.35
Gram-positive bacteria	38	29.92
*Staphylococcus epidermidis*	10	26.32
Other CoNS	9	23.68
*Streptococcus*	7	18.42
*Staphylococcus aureus*	6	15.79
*Enterococcus*	4	10.53
*Micrococcus*	1	2.63
*Bacillus*	1	2.63
Fungus	7	5.51
*Candida albicans*	7	5.51
Total	127	100.00

VAP, ventilator-associated pneumonia; CoNS, coagulase negative staphylococcus.

**Table III:** Antimicrobial resistance patterns of common gram-negative bacteria isolated from the sputum of children with VAP.

Drugs	Pathogens (drug resistance rate, %)				
	*Acinetobacter baumannii* (21 strains)	*Escherichia coli* (17 strains)	*Stenotrophomonas maltophilia* (17 strains)	*Klebsiella pneumoniae* (12 strains)	*Pseudomonas aeruginosa* (11 strains)
Cefoperazone/sulbactam	57.14	0.00	0.00	0.00	0.00
Cephazoline	100.00	100.00	100.00	100.00	100.00
Cefoxitin	100.00	50.00	35.29	50.00	100.00
Amikacin	80.95	11.76	100.00	0.00	0.00
Levofloxacin	42.86	35.29	0.00	25.00	0.00
Cefuroxime	100.00	82.35	100.00	100.00	100.00
Ceftazidime	100.00	70.59	100.00	100.00	100.00
Ampicillin	100.00	100.00	100.00	100.00	100.00
Cefpiramide	100.00	76.47	100.00	100.00	0.00
Gentamicin	100.00	82.35	100.00	50.00	0.00
Imipenem	100.00	0.00	70.59	0.00	0.00
Meropenem	100.00	0.00	100.00	0.00	72.73
Cefotaxime	61.90	70.59	100.00	41.67	0.00
Piperacillin/tazobactam	80.95	0.00	0.00	25.00	0.00

VAP, ventilator-associated pneumonia.

**Table IV:** Antimicrobial resistance patterns of staphylococci isolated from the sputum of children with VAP.

Drugs	Pathogens (drug resistance rate, %)		
	*Staphylococcus epidermidis* (10 strains)	Other CoNS (9 strains)	*Staphylococcus aureus* (6 strains)
Clindamycin	60.00	44.44	50.00
Linezolid	0.00	0.00	0.00
Ampicillin/sulbactam	100.00	77.78	0.00
Gentamicin	40.00	44.44	33.33
Oxacillin	100.00	88.89	0.00
Rifampicin	20.00	11.11	0.00
Sulfamethoxazole	90.00	55.56	66.67
Vancomycin	0.00	0.00	0.00
Moxifloxacin	0.00	0.00	0.00
Erythromycin	90.00	55.56	83.33
Furantoin	0.00	0.00	0.00
Levofloxacin	0.00	33.33	33.33
Penicillin-G	100.00	88.89	83.33
Tetracycline	40.00	22.22	0.00

VAP, ventilator-associated pneumonia; CoNS, coagulase negative staphylococcus.
